# The Effects of Extender Energetic Substrate Type on Goat Sperm Stored at 17 °C

**DOI:** 10.3390/biology14070782

**Published:** 2025-06-27

**Authors:** Sabrina Gacem, Eva Mocé, Carmen Gozalbo, Marta Albuixech-Benetó, Inés C. Esteve, Amparo Martínez-Talaván, Miguel A. Silvestre

**Affiliations:** 1 Departamento de Biología Celular, Biología Funcional y Antropología Física, Universitat de València, 46100 Valencia, Spain; swp.sabrina.gacem@gmail.com (S.G.); carmengozalbo200140@gmail.com (C.G.); martaalbuixech1@gmail.com (M.A.-B.); 2 Centro de Investigación y Tecnología Animal, Instituto Valenciano de Investigaciones Agrarias, 12400 Castellón, Spain; esteve_ine@gva.es (I.C.E.); martinez_amptal@gva.es (A.M.-T.)

**Keywords:** pyruvate, lactate, fructose, glucose, sperm, goat

## Abstract

Artificial insemination in goats commonly relies on refrigerated semen doses, yet the optimal energetic substrate to support sperm metabolism remains unclear. This study aimed to evaluate the effects of different energetic substrates on goat buck sperm metabolism and motility when refrigerated at 17 °C. Semen from six Murciano-Granadina male goats were collected and diluted in PBS supplemented with 35 mM of either glucose, fructose, pyruvate, or lactate in the first experiment. In the second experiment, the effects of varying concentrations of pyruvate and/or glucose, NaCl supplementation, and the osmolarity on sperm quality parameters were assessed. Semen was stored at 17 °C for 48 h and evaluated for motility using the CASA system, as well as for viability, mitochondrial membrane potential, and mitochondrial ROS by flow cytometry. The results show that pyruvate and lactate extenders outperformed the others, preserving higher total motility, progressivity, and viability of spermatozoa over 48 h, even at a concentration lower than 35 mM, as in the case of pyruvate. In contrast, glucose had a detrimental effect on sperm quality, reducing viability and healthy population rates while increasing motility, especially at higher concentrations. NaCl supplementation and osmolarity had no significant effect on any of the sperm quality parameters. In conclusion, pyruvate maintains a higher quality and motility of sperm stored at 17 °C in PBS in comparison with a glucose-supplemented extender.

## Simple Summary

Understanding how energy sources influence sperm performance could lead to optimized semen preservation techniques and ultimately benefit reproductive technologies and livestock breeding programs. Currently, extenders formulated for goat buck sperm primarily contain glucose as the main energy source. However, despite recent efforts to optimize extenders, no significant successful improvement in artificial insemination with cooled semen stored for over 24 h has been reported. The current study aimed to investigate the effect of pyruvate, lactate, glucose, and fructose on sperm during refrigeration at 17 °C. Pyruvate proved to maintain better sperm quality parameters than the other substrates after 48 h of storage at 17 °C in PBS.

## 1. Introduction

Artificial insemination (AI) plays an important role in the breeding programs of dairy goats by enabling the testing of male candidates, the spread of genetic gains obtained by the dissemination of seminal doses from elite males, by establishing strong links between herds that allow for male candidates to be properly tested, and by enabling the evaluation of the genetic value of the animals composing the population [1,2]. However, AI is not broadly used for this species and remains low in many dairy goat breeds if compared with other species [2], which affects breeding programs by slowing down their advancement.

Most of the AI for this species is performed with refrigerated semen doses used on the same day the semen is collected [3]. With these doses, fertility remains high if the semen is used in the first 5–8 h after being produced [3]. The short period that liquid stored doses keep their fertilizing ability hinders the delivery of doses to herds located far away from the AI centers, and also impedes the use of transport agencies to send the seminal doses. Therefore, if the long-term storage of refrigerated goat buck sperm is achieved, its impact on the use of AI in this species will be significant. Compared to other species in which refrigerated semen is used for AI (such as with porcine semen), the availability of extenders for the liquid storage of goat sperm is very limited, and they are usually extenders developed for bull sperm. Thus, some authors have highlighted the lack of consistent studies on the optimal composition of an extender for the long-term liquid storage of goat buck sperm [1].

Sperm are cells with an energetic metabolism and are very active, and the energy produced is used for motility and the maintenance of membrane integrity, among other functions [4]. For long-term sperm storage in a liquid state, an extender must include ingredients adapted to the sperm’s physiology [5]. For this reason, energetic substrates are one of the essential components of semen extenders. Traditionally, extenders contained glucose or fructose as energetic substrates because glycolysis was considered the main energetic metabolic pathway in sperm [4]. However, there is increasing evidence showing that the principal metabolic pathway for producing energy differs between species or according to the sperm’s physiological condition [6]. Thus, while mice, humans, and other primate species mainly use the glycolysis pathway for energy production [7], in other species like horses sperm preferentially use oxidative phosphorylation (OXPHOS) to maintain their functions [5], at least at physiological temperatures [7]. Bull sperm also depends on this pathway to support the capacitation process [6]. According to a recent study, goat buck sperm uses both pathways to support sperm motility [8].

Nevertheless, there is increasing evidence that sperm have metabolic plasticity, and their metabolic preference may vary according to the surrounding environment and physiological events (for a review, see [4]). Indeed, research has shown that each individual process and event (motility, capacitation, or acrosome reaction) or storage temperature (body temperature vs. 4 °C) requires a different substrate and metabolic pathway [7,9]. In caprine sperm, recent reports suggest that the OXPHOS pathway plays a more important role in supporting sperm motility during liquid storage [10].

The extenders currently formulated for goat buck sperm contain exclusively fructose or glucose as their main energetic substrates, although the growing knowledge on goat buck sperm metabolism (despite being incipient) calls for an update of the extenders’ formulation. In recent years, research has been carried out to optimize the extender used [3,11,12,13,14,15], but to date there have been no optimal results of AI with cooled semen doses stored for longer than 24 h. Thus, more studies should be conducted to determine the most adequate energetic substrate in this species, since research in this area is limited. In addition, the choice of energy metabolic pathways for ATP synthesis and sperm motility during cooled storage could be influenced by a variety of energy conditions, but how these conditions affect goat sperm is unknown.

Thus, the aim of the current study is to determine the effect of different energetic substrates in phosphate-buffered saline (PBS)-based extenders on goat buck sperm quality during cooled storage.

## 2. Materials and Methods

### 2.1. Reagents and Media

The chemicals and media used were all purchased from Merck (Darmstadt, Germany), unless otherwise specified. The base semen extender consisted of PBS (D5652). For all experiments, NaCl was added to different media to adjust extender osmolality. The extenders’ compositions are detailed in Table 1.

### 2.2. Semen Collection and Processing

Males (*n* = 6) belonging to the Murciano-Granadina goat breed were used during this study. The animals were reared in the Centro de Tecnología Animal from Instituto Valenciano de Investigaciones Agrarias (CITA; 39°52′ N, 0°30′ W; Segorbe, Spain). They were housed in pens with access to water and straw ad libitum. Moreover, they were supplied with 1 kg/day of concentrated feed (17% crude protein, 11.6% crude fiber, and 4.5% crude fat) per male. The CITA center has obtained approval for the collection, processing, preservation, and storage of semen doses (registration number: ES10003). The protocols for the semen collection, care, and housing of the male goats at the breeding center complied with the European standards for the care and use of animals for scientific purposes. Semen was collected twice per week from mature, clinically healthy bucks aged between 2 and 7 years. According to legislation (RD 118/2021; BOE, 2021), semen collection with an artificial vagina is considered a routine husbandry practice and, for this reason, does not require ethical approval. Semen was collected using an artificial vagina [16] from March to May. After ejaculate collection, an aliquot of semen was taken from each ejaculate (Experiment 1) or pool (Experiment 2) of semen, diluted with PBS, and used to analyze sperm motility. Only semen with a motility higher than 40% was used for this experiment. Seminal plasma was washed in accordance with the protocol described by [17]. Briefly, the semen was diluted up to 10 mL in PBS and centrifuged for 15 min at 500× *g* at room temperature (~22 °C), and the supernatant was discarded. Then, the pellet was resuspended in 10 mL of PBS and centrifuged for a second time. After this, the supernatant was removed, and the pellet was homogenized in the remaining PBS. Later, the volume was determined and the concentration was measured in accordance with the protocol described by [17] using a photometer (Accucell, IMV Technologies, L’Aigle, France).

After that, aliquots were taken and were diluted in the corresponding extender (see below in Experimental Design) to a final concentration of 560 × 10^6^ sperm/mL [12] and were cooled from 20 to 17 °C in a programmable water bath (Julabo GmbH, Seelbach, Germany) at a rate of −0.18 °C/min. All the preparations were stored at 17 °C for 48 h. Analyses of motility, viability, mitochondrial activity, and oxidation status were performed at 2, 24, and 48 h.

### 2.3. In Vitro Sperm Quality Evaluation

#### 2.3.1. Sperm Motility Analysis

Sperm motility was assessed using the new generation of the CASA-Mot system with artificial intelligence to recognize sperm from images (AI Station v1.2; Sperm Analysis Technologies S.L, Buñol, Spain). Following the manufacturer’s recommendation, an aliquot of 3 μL of semen suspension was loaded into capillary-loaded counting chambers (Spermlide^®^ 20 μm; Sperm Analysis Technologies SL, Buñol, Spain). Throughout the analysis, the temperature was maintained at 37 °C by means of a temperature-controlled stage. Video captures were recorded at 100 frames per second using a FLIR digital camera (2048 × 1536 resolution, 1/2″ sensor) mounted on a microscope equipped with a 10× negative phase contrast objective (NA 0.25). Three to five fields were recorded for each sample to achieve a final minimum count of 500 spermatozoa. Videos were recorded for 1 s for each field. The sperm kinematic parameters assessed by CASA included curvilinear velocity (VCL, µm/s) measured in a point-to-point reconstitution of the sperm trajectory; straight-line velocity (VSL, µm/s), defined by the straight line between the first and last point on the track; and average trajectory velocity (VAP, µm/s), linearity (LIN: VSL/VCL × 100, %), and straightness (STR: VSL/VAP × 100, %). In addition, two sperm motility ratios, expressed as percentages, were calculated—the total motility (TM) and progressive motility (PM) of motile sperm.

#### 2.3.2. Flow Cytometry Analysis

All flow cytometry assessments were conducted in the core facility for cell culture and flow cytometry (FC) in the Central Service for Experimental Research (SCSIE) at the University of Valencia. The samples were analyzed using a BD LSRFortessa™ SORP (Beckton Dikinson, San Jose, CA, USA) flow cytometer equipped with five lasers emitting UV wavelengths at 355 nm, blue at 488 nm, yellow-green at 561 nm, violet at 405 nm, and red at 640 nm. The system was controlled using FACSDiva 8 software. A minimum of 10,000 cells per replicate was recorded, and the flow rate was maintained at 500–1500 cells/s. Sperm parameters of viability, mitochondrial membrane potential (hMMP), and mitochondrial reactive oxygen species production (mROS) were evaluated with a triple staining of the fluorescents DAPI/Mitotracker Deep Red/MitoSOX™ Red following the protocol described by Gacem et al. [18]. For all fluorochromes, cells with a high intensity were considered positive and those with a low intensity negative. Briefly, DAPI staining (D9542; Merck Life Science S.L.U., Madrid, Spain) was used to assess sperm viability, considering that DAPI-positive and -negative cells are dead and live cells, respectively. MitoTracker^®^ Deep Red (MTDR; M22426; Invitrogen™, Fischer Scientific, S.L; Madrid, Spain) was used to assess the mitochondrial membrane potential of the cells, considering that MTDR-positive and -negative cells are sperm with high and low mitochondrial membrane potential, respectively. MitoSOX™ Red (M36008; Invitrogen™, Fischer Scientific, S.L; Madrid, Spain) was used to assess the amounts of superoxide anion radical produced by the mitochondria. MSOX-positive and -negative spermatozoa were considered to be sperm with high and low amounts of superoxide. DAPI-positive, MTDR-positive, MSOX-negative cells were considered “healthy”. The semen samples were incubated with a mix of fluorochromes in PBS for 15 min at 37 °C in the dark and analyzed by FC.

### 2.4. Experimental Design

To achieve the objective, two experiments were carried out. The compositions of the PBS-based extenders used in Experiments 1 and 2 are shown in Table 1.

#### 2.4.1. Experiment 1: Effects of Glucose, Fructose, Pyruvate, or Lactate as Energetic Extender Substrates on Sperm Quality Following Storage at 17 °C for 48 h

In this experiment, we determined the individual effect of the energetic substrate on sperm metabolism. For this, individual ejaculates (*n* = 8) from 6 males were used. After seminal plasma removal, each of the samples was divided into five aliquots: one of them was diluted in a PBS (control group; N) and the other aliquots were diluted in PBS supplemented with 35 mM of glucose (G), fructose (F), pyruvate (P), or lactate (L). In addition, to adjust extender osmolality to 340 mOsm/kg in all the treatments, NaCl was added to the N, G, and F groups. After dilution, they were refrigerated to 17 °C and stored at this temperature for 48 h. Sperm quality parameters were evaluated at 2 h, 24 h, and 48 h of storage.

#### 2.4.2. Experiment 2: Effects of Glucose, Pyruvate, NaCl Supplementation, and Osmolarity on Sperm Quality Following Storage at 17 °C for 48 h

In this experiment we studied the effects of different concentrations of glucose and pyruvate either alone or in combination, as well as the effect of osmolarity and NaCl supplementation on goat sperm quality when stored at 17 °C for up to 48 h. For this, 6 pools of semen collected from 5 males were used. Each pool comprised semen from two males. After seminal plasma removal, each of the samples was divided into nine aliquots and these were diluted in PBS to which different supplementations were added (Table 1): PBS supplemented with glucose at 35 mM or 70 mM (G35, G70), PBS–pyruvate at 35 mM (P35), PBS–pyruvate at 18 mM (P18), PBS–glucose at 35 mM and –pyruvate at 18 mM (G35/P18), PBS–glucose at 18 mM and –pyruvate at 9 mM (G18/P9), PBS–glucose at 35 mM and –NaCl at 18 mM (G35/N18), PBS–glucose at 18 mM and –NaCl at 9 mM (G18/N9), or PBS–pyruvate at 18 mM and –NaCl at 9 mM (P18/N9). Thus, extenders with different osmolarities were made (high 321–348 mOsm/kg vs. low: 298–313 mOsm/kg). After dilution, they were refrigerated to 17 °C and stored at this temperature for 48 h. Sperm quality was evaluated at 2, 24, and 48 h of storage.

### 2.5. Statistical Analysis

Statistical analyses were performed using a statistical software package (IBM^®^ SPSS^®^ 28.0.1.1 for Windows; IBM corp., Armonk, NY, USA). Data were tested for normality (Shapiro–Wilk). The variables that did not follow a normal distribution were transformed by calculating the arcsine of the square root for percentage variables or the logarithm for non-percentage variables. The variables were analyzed using Generalized Linear Models (GLMs). For Experiment 1, we analyzed one model that included the effects of energetic substrate (N, G, F, L, and P), time (0, 24, and 48 h), and double interactions for all studied variables. For Experiment 2, three models were used. The first one included the effect of the experimental group (G35, G70, P35, P18, G35/P18, G18/P9, G35/N18, G18/N9, and P18/N9) and time (0, 24, and 48 h) on total motility and viability. The second one included the effects of time, energetic substrate (G: groups with only glucose, P: groups with only pyruvate, M: groups with combinations of glucose and pyruvate), osmolarity (H, L), the presence of NaCl, and double interactions in the model for all variables. The last model included the effect of concentration (70, 35, 18 mM) and energetic substrate, glucose or pyruvate (G or P), for all variables. A probability of *p* < 0.05 was considered statistically different and the data are shown in the tables and figures as the mean ± the standard error of the mean (SEM).

## 3. Results

### 3.1. Experiment 1: Effects of Glucose, Fructose, Pyruvate, or Lactate as Energetic Extender Substrates on Sperm Quality Following Storage at 17 °C for 48 h

**Figure 1:**
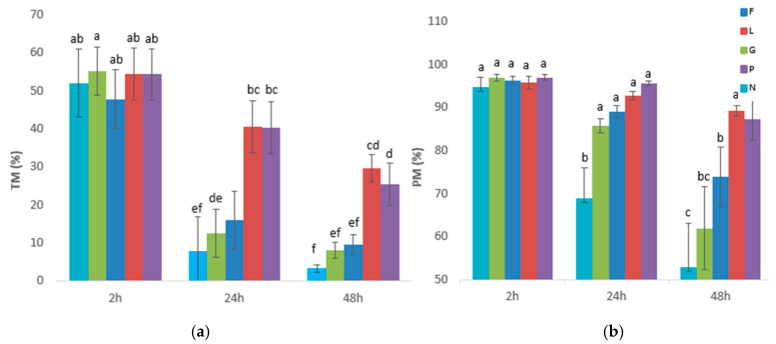

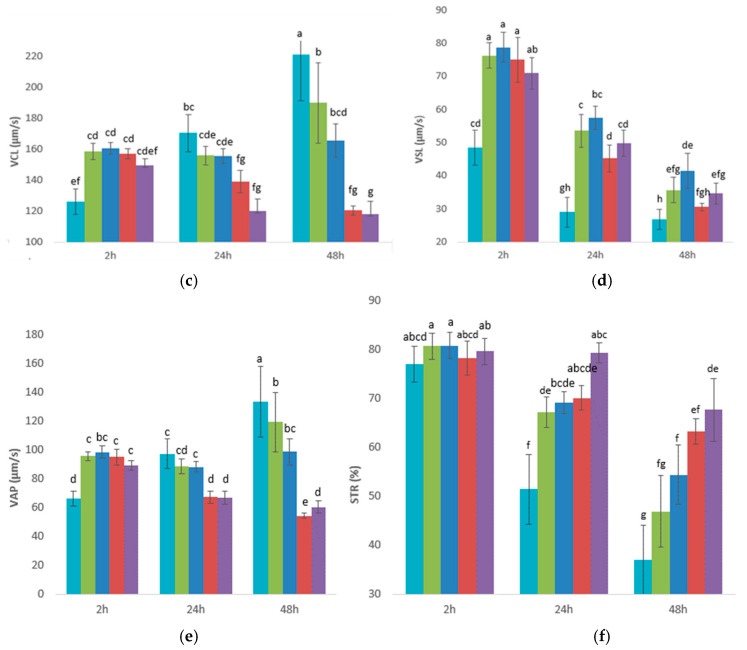
The effects of glucose, fructose, pyruvate, or lactate on spermatozoa motility when stored at 17 °C. The goat sperm samples were stored in PBS containing glucose (G), fructose (F), lactate (L), or pyruvate (P) and the control was stored without an energy substrate but was supplemented with NaCl (N). Motility parameters were recorded by CASA at 2, 24, and 48 h of incubation at 17 °C. (**a**) Total motility (TM). (**b**) Progressive motility (PM). (**c**) Curvilinear velocity (VCL). (**d**) Straight-line velocity (VSL). (**e**) Average path velocity (VAP). (**f**) Straightness index (STR). (^a–h^) indicate differences between interaction treatments (energetic substrates × storage time) (*p* < 0.05). The results are expressed as the mean ± standard error.

The interaction (energetic substrate x storage time) was significant. In Figure 1, the results presented correspond to the variables of sperm motility observed for the interaction (energetic substrates x storage times at 17 °C). For TM (Figure 1a) and PM (Figure 1b), all the extenders presented similar values at 2 h (around 55% and 95%, respectively, for TM and PM). However, significant differences between the energetic substrates arose after 24 h of storage at 17 °C. Thus, the samples preserved in the P and L extenders exhibited a higher TM than the samples preserved in the N, G, or F extenders (*p* < 0.05). In addition, the PM decreased after 24 h only in the samples diluted in the N extender, but after 48 h more differences appeared between the energetic substrates. Thus, while P and L exhibited the highest values for PM after 48 h (values similar to those they exhibited after 2 h), the PM decreased significantly to 75% in the F extender and 62% in G after 48 h. In addition, the N extender showed the lowest sperm PM (Figure 1b).

Regarding sperm kinetics, the N extender presented the lowest value for VAP (Figure 1e) at 2 h and was significantly different compared to the values exhibited by the other extenders. From then on, the samples diluted in the N extender exhibited a change in their kinematics since at 24 h they exhibited similar values to those observed in the G and F groups and higher than the values presented by the L and P extenders. Moreover, after 48 h of storage, VAP increased significantly in the N extender, thus exhibiting higher values than those presented by the samples preserved in the other extenders supplemented with energetic substrates. As for the G extender, the sperm retained VAP at 24 h compared to 2 h and increased it significantly at 48 h. When sperm was diluted in the F extender, the VAP value remained constant during storage, while with the L extender, VAP decreased significantly as storage time progressed from 2 to 48 h, reaching the lowest value at 48 h. Regarding the P extender, the VAP dropped significantly after 24 h, but this value did not decrease further after 48 h of storage. In broad terms, the samples behaved similarly regarding VCL (Figure 1c) at all the points of the analyses, only showing slight differences. Thus, after 48 h of storage and in the absence of an energetic substrate (the N extender), the sperm exhibit their highest curvilinearity compared to the other extenders supplemented with energetic substrates. For VSL (Figure 1d), the behavior at 2 h was similar to those at other velocities in which the N extender exhibited the lowest value compared to the other extenders. After 24 h of storage, the VSL decreased significantly in all extenders and more differences arose between the extenders supplemented with the energetic substrates. Thus, the sperm stored in the P extender showed a similar VSL to the sperm diluted in the other extenders (G, F, and L). However, the sperm stored in the L extender exhibited a lower VSL than the sperm stored in the G and F extenders (*p* < 0.05). At 48 h, the VSL decreased further in all of the extenders, except for the samples diluted in the N extender, which exhibited a similar VSL to the one they presented at 24 h. However, at this point the differences between the energetic substrates disappeared since all of them exhibited similar VSL values. Samples diluted in the N extender always exhibited the lowest VSL values at all storage durations (2, 24, and 48 h) compared to the other extenders. Sperm straightness (STR; Figure 1f) was similar for all of the extenders at 2 h. This value decreased after 24 h of storage in all of the extenders except for the samples kept in the P extender, which exhibited similar values at 2 and after 24 h. In addition, at 24 h the N samples exhibited a lower STR than the other extenders, and the P extender exhibited the highest value, although it was similar to those exhibited by the samples diluted in the F extender. The STR decreased significantly after 48 h in all of the extenders, reaching the lowest values in the N extender and the highest value in the P extender.

**Figure 2:**
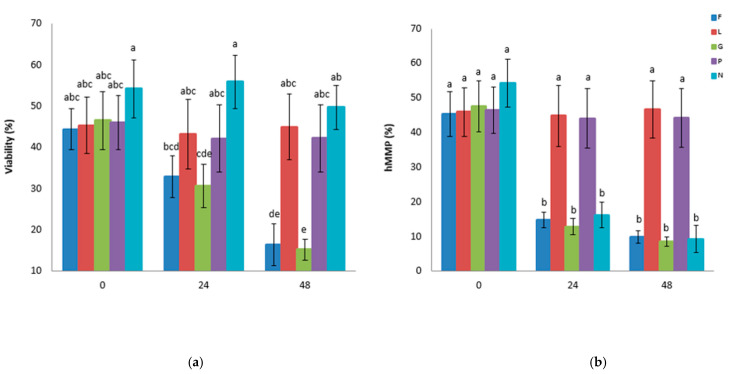

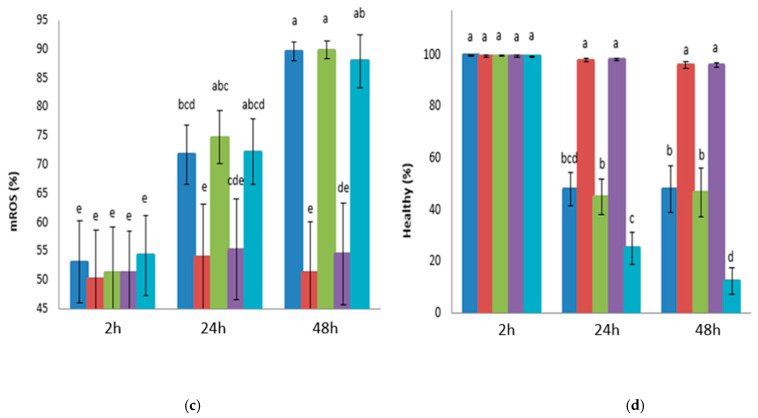
The effects of glucose, fructose, pyruvate, or lactate on spermatozoa viability, mitochondria membrane potential, oxidative stress reaction, and healthy population when stored at 17 °C. Goat sperm was stored in PBS containing glucose (G), fructose (F), lactate (L), or pyruvate (P), and the control was stored without an energy substrate but was supplemented with NaCl (N). (**a**) Viability, (**b**) sperm with high mitochondrial membrane potential (hMMP), (**c**) reactive oxygen species production (mROS), and (**d**) sperm having high mitochondrial membrane potential and low oxidative stress (healthy). (^a–e^) indicate differences between interaction treatments (energetic substrates × storage time) *(p* < 0.05). The results are expressed as the mean ± standard error.

The results for the variables of the FC analyses of the interaction between the energetic substrate and the storage time at 17 °C are shown in Figure 2. Regarding the viability rate, both the L and P extenders exhibited significantly higher viability than the G and F extenders at 24 and 48 h of storage (*p* < 0.05) (Figure 2a). It can be observed that spermatozoa in the L and P extenders maintained the same hMMP and underwent significantly less oxidative stress after 48 h of storage. However, the hMMP in F, G, and N decreased to less than 20% with a higher mROS (Figure 2b,c). The same effect was observed in the percentage of healthy sperm (Figure 2d).

### 3.2. Experiment 2: Effects of Glucose, Pyruvate, NaCl, Osmolarity, and Energetic Substrate Concentration on Sperm Quality Following Storage at 17 °C for 48 h

The results of the experimental groups G35, G70, P35, P18, G35/P18, G18/P9, G35/N18, G18/N9, and P18/N9 during refrigerated storage, for all variables, are provided in Supplementary Tables S1 and S2. The results of NaCl supplementation, osmolarity, and extenders (G, M, and P) on all the variables are presented in Table 2, Table 3 and Table 4. NaCl supplementation did not have any significant effect on any of the sperm quality parameters evaluated (Table 2 and Table 3). Osmolarity only influenced the VSL; only the samples diluted in the lowest osmolarity media exhibited significantly higher VSL values (Table 2). The extender did not have any effect on sperm TM, PM, or kinetic parameters (Table 2), but marked differences were observed between extenders for viability, hMMP, mROS production, and healthy sperm (Table 3). Except for the hMMP, which was similar for the G and M extenders, significant differences (*p* < 0.05) were observed between all the extenders for all the variables studied. Thus, samples diluted in the extenders that included only pyruvate as an energetic substrate always exhibited higher values, while the samples diluted in the extenders that contained glucose exhibited the lowest values for viability, hMMP, and healthy sperm. In addition, the lowest mROS values were observed in the P extender and the highest mROS values were exhibited by the samples diluted with the G extender. The extender in which P and G were combined (the M extender), always exhibited intermediate values.

**Figure 3:**
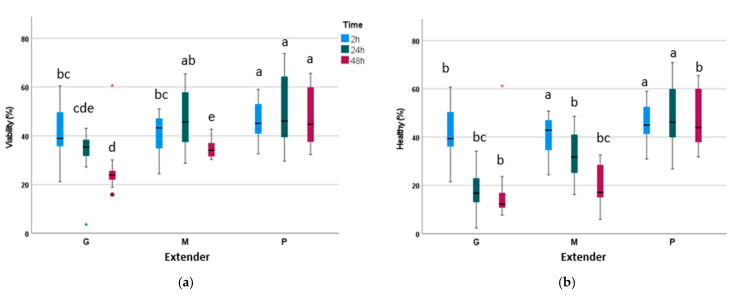
The effects of glucose and/or pyruvate on sperm viability and healthy sperm (sperm exhibiting high mitochondrial membrane potential and low oxidative stress). (**a**) Viability, (**b**) healthy: sperm having high mitochondrial membrane potential and low oxidative stress. G: an extender containing glucose; M: an extender containing glucose and pyruvate; P: an extender containing pyruvate. Blue: time 2h; Green: time 24h; Red: time 48h. (^a–e^) indicate differences between interaction treatments (time x extender) (*p* < 0.05).

None of the triple interactions were significant (Table 4). However, a significant interaction in terms of Time × Osmolarity was observed for VSL, while Time × Extender resulted in significant differences for viability, high mitochondrial membrane potential (MMP), mROS production, and healthy sperm. The results for the interaction of Time × Extender are shown in Figure 3. The P extender exhibited the highest values for viable sperm (Figure 3a), values which were maintained over 48 h of storage at 17 °C. However, when glucose was added as an energetic substrate alone or in combination with pyruvate, sperm viability was maintained for 24 h but decreased significantly after 48 h of storage. The lowest viability rates at 24 and 48 h corresponded to the extender containing only glucose. The healthy sperm population (Figure 3b) was highest in the pyruvate media at 2 h and 24 h and decreased significantly after 48 h.

Changes in glucose and pyruvate concentration affected the sperm quality parameters (Table 5). Regarding the glucose, samples diluted in extenders containing 35 mM of G exhibited higher sperm velocity, TM, and PM than the samples diluted with 18 and 70 mM of G (*p* < 0.05). However, sperm viability and the healthy population percentage were higher, while mROS was lower in the 18 mM G extender. In the P extender the TM, PM, and all kinematic parameters were higher (*p* < 0.05) at the highest pyruvate concentration (35 mM) than the other glucose concentrations. Similarly to the glucose, the opposite was observed for the parameters evaluated with FC since, in that case, the sperm quality was higher for the lower pyruvate concentration (18 mM).

## 4. Discussion

The choice of an appropriate extender is critical for long-term sperm preservation, as it can significantly affect sperm motility and viability when semen is stored refrigerated. Traditionally, glucose has been widely used as an energetic substrate in extenders to provide physiological osmolarity and as an energy source for the sperm of many species [5,19]. In goats, glucose and fructose are commonly used in semen extenders for AI, regardless of the extender used—i.e., whether citrate [20,21,22,23,24] or milk [17,25,26,27]. However, recent studies have highlighted potential conflicts between classic extender formulations and the evolving understanding of sperm metabolism [4,6], suggesting the need to reevaluate and optimize extender components. Therefore, this study aimed to investigate the effect of different energetic substrates on goat semen quality after 48 h of storage in PBS at 17 °C.

In this study, semen was diluted in PBS at 17 °C. Although PBS is not an optimal diluent to preserve sperm, it was selected as it lacks energy substrates, thus providing a controlled medium to evaluate sperm metabolism without the influence of other energetic substrates. The use of extenders containing additional substrates could lead to metabolic competition, potentially altering sperm performance and confounding the interpretation of the results. Also 17 °C was used as the temperature of conservation since goat sperm demonstrates superior quality when preserved in PBS at 15 °C compared to 5 °C [10], whereas the milk extender is usually more effective for preservation at lower temperatures [28]. Semen quality is known to decline during extended refrigeration (24–48 h), as observed in our initial experiment and our previous works [28]. Sperm quality and viability progressively decreased over time, reaching their lowest levels after 48 h when stored in various extenders. Spermatozoa can derive energy from exogenous substrates or utilize endogenous reserves, depending on metabolic preference between species, oxygen availability, energy demands, and the composition of metabolic substrates in the extender [29,30]. In the case of goat sperm, it uses both pathways when incubated at 37 °C [8]. When glucose and fructose are in the extender, spermatozoa uses them via glycolysis producing ATP in the cytoplasm, regardless of the oxygen availability in the environment [31]. Glucose enters sperm through glucose transporters, converts to pyruvate, and produces two ATP. While fructose is converted to intermediates like DHAP and glyceraldehyde-3-phosphate that feed into glycolysis [6,29]. Glycolysis occurs in the flagellum, ensuring a localized energy supply for movement. The enzymes used in this last metabolic pathway are affected by lower temperatures when in liquid storage [32]. When pyruvate is readily metabolizable in the extender, it directly enters the electron transport chain in the mitochondria localized in the mid-piece and converts to acetyl-CoA, which fuels the Krebbs cycle for ATP production (32 ATP) in a process called OXPHOS [30]. In the absence of pyruvate, lactate is converted to pyruvate which is the necessary product for OXPHOS [6,29]. Studies have shown that high sperm motility and straighter swimming paths are strongly associated with energy production primarily driven by OXPHOS [33,34], which correlates to our findings in the first experiment in the presence of pyruvate and lactate. In the first experiment, sperm stored in extenders containing pyruvate or lactate maintained significantly elevated in vitro sperm quality parameters (higher motility, progressivity, viability, hMMP, healthy population, and lower ROS production) after 48 h of storage compared to the other extenders. To the best of our knowledge, this is the first study that jointly compares the effect of different energy substrates on goat sperm metabolism when refrigerated at 17 °C. However different studies have been conducted for different species in which pyruvate increased total motility and progressivity in human and stallion sperm [35,36,37].

The positive impact of pyruvate as an energetic substrate that not only enhances sperm viability, hMMP, and healthy population but also decreases ROS production could be attributed to pyruvate’s antioxidant role, it having been recognized for its ability to destroy the H_2_O_2_ generated by sperm and to protect against oxidative damage [38,39]. Even if it is well-known that the production of ROS is higher from oxidative phosphorylation that uses pyruvate [40,41,42,43], recent studies have nonetheless found significant positive correlations between ROS and overall sperm motility [44,45]. This antioxidant capacity was described for the males of different species such as rams [46], bucks [47], bulls [48], stallions [49], and boars [50]. In addition, pyruvate was added during IVF as it enhances fertility by improving sperm function in humans [35]. Also, exogenous pyruvate accelerates glycolysis by regenerating NAD^+^ after its conversion to lactate, acting independently of mitochondrial respiration in anaerobic conditions [35,37]. We can suggest that pyruvate improves sperm motility and quality after long storage in refrigerated conditions. However, the concentration of pyruvate, as shown in the last experiment, should not be neglected since a high-pyruvate concentration was associated with higher mROS generation, explained by the higher motility and velocities, which in turn produce more mROS. However, Qiu et al. [10] found that using 10 mM pyruvate achieved better progressivity after 7 days of storage when compared to other concentrations (0, 5, or 30 mM). Lower concentrations were used in other studies of human and stallion semen, showing 5 mM higher TM and progressivity [35,51].

Similarly to pyruvate, lactate also achieved better sperm quality than glucose or fructose. However, the VCL and TM were higher in lactate than pyruvate while pyruvate achieved greater straightness and motility. Almost the same result was described in stallion sperm when the semen was short term incubated at 37 ºC in extenders supplemented with 5.5 mM of pyruvate and lactate [45]. Even if the metabolisms of pyruvate and lactate are strictly correlated, it is likely that pyruvate has a faster metabolism in mitochondria than lactate [7]. Lactate was also shown to have antioxidant activity by scavenging free radicals in an in vitro cell culture [52]. Moreover, the physiological environment of the oviduct, a critical site for fertilization, further underscores the importance of lactate. During the estrus period, lactate concentrations in the oviduct are significantly higher than glucose levels, whereas both substrates are present in lower concentrations in the uterus [29,53]. This elevated lactate availability in the oviduct reflects its specialized environment, which meets the high metabolic demands of sperm, aligning with the observed benefits of lactate-rich extenders.

In the second experiment, the proportion of healthy sperm was significantly higher in the P group compared to the G and M groups, which is in accordance with our first experiment where pyruvate alone had better results for sperm metabolism. Higher TM was observed in extenders containing pyruvate, including in the N extender without energetic substrates, compared to glucose-based extenders. This finding suggests that glucose negatively impacts sperm viability, hMMP, and increases ROS production in our conditions. These observations were confirmed when we analyzed how glucose concentration in the extender affects sperm quality parameters. Lower glucose concentrations were associated with a higher healthy population percentage with high viability and mitochondrial activity and lower ROS production. In contrast, increasing glucose concentrations negatively impacted sperm metabolism. However, sperm velocity (VCL, VSL, and LIN) and progressivity were higher when glucose concentration increased to 35 mM, thus explaining the elevated ROS production, which negatively affects sperm membranes and viability. Our finding was in accordance with previous studies [10,54,55] which also observed increased ROS levels. However, motility rates were reduced compared to our finding; this could be explained by the highest glucose concentration (higher than 172 mM). Additionally, Xu et al. [15] found higher TM in goat sperm stored in low-glucose extenders such as Androhep and Zorlesco. Similarly, Gororo et al. [13] demonstrated that eastern African goat sperm preserved in a low-glucose media exhibited significantly greater motility and viability compared to those preserved in high-glucose extenders.

Other studies have also demonstrated the effective role of pyruvate when added to glucose-based extenders, like in stallions, as in the case of Martín-Cano et al. [56] who reported that adding pyruvate to PBS containing glucose enhanced sperm viability at 37 °C compared to glucose-only extenders. Furthermore, Darr et al. [45] demonstrated that pyruvate increased mitochondrial activity and superoxide anion levels in stallion sperm. Yet, contrasting findings were reported by Hernández-Avilés et al. [7], who observed better sperm quality (viability and motility) in stallion sperm incubated with glucose. The differences observed in these studies may be attributed to the type of extender (skimmed milk) and the temperature conditions used, which may affect the metabolic pathway choice. The observed detrimental effect of glucose on goat sperm can be explained by the intracellular acidosis resulting from high glucose levels [57,58]. Additionally, glucose’s toxic effect on sperm could be related to the excessive production of oxaldehydes such as glyoxal and methylglyoxal during glycolysis [54]. Furthermore, glucose was shown to decrease the expressions of 5′-AMP-activated protein kinase, a key regulator of energy homeostasis. This effect likely occurs through the stimulation of ATP production or the inhibition of ATP consumption, which may impair the sperm’s ability to maintain energy balance [55].

Regarding the N extender, after 48 h of storage, high VCL and VAP, along with low TM and PM, were observed when compared to other extenders supplemented with energetic substrates. This motility pattern (high VCL) may reflect the presence of dying spermatozoa exhibiting erratic and non-progressive movements [12,59,60].

In Experiment 2, NaCl was used to balance osmolarity between the diluents and the potential impact of NaCl supplementation on goat sperm was investigated to determine whether its use in the experiment could influence the results. It was found that NaCl supplementation had no significant effect on sperm metabolism or motility. Likewise, the effect of osmolarity on sperm was investigated and no significant differences were observed, which can be attributed to the relatively small variation in osmolarity between the groups (±50 mOsm). Sperm cells are known to survive in extenders with osmolarities ranging from 425 to 525 mOsm [61].

## 5. Conclusions

Selecting the appropriate energetic substrate with the proper concentration for short-term refrigerated sperm storage is crucial, as it directly impacts sperm motility and quality. While glucose has long been used, it has been shown to reduce sperm quality over time by negatively affecting the sperm membrane and metabolism, even when pyruvate was added. In contrast, using the appropriate concentration of pyruvate or lactate demonstrated a better preservation of sperm quality, making them a more effective choice for maintaining sperm quality during short-term storage at 17 °C in PBS. Nevertheless, when using different extenders or when relying on refrigeration at lower temperatures and more complex extenders, the effect of these substrates should be investigated since these conditions may affect the sperm metabolism differently.

## Figures and Tables

**Table 1:** Composition of PBS-based extenders used in Experiments 1 and 2.

PBS-Based Extender	Osmolarity Group	D(+) Glucose Anhydrous (mM)	D(-) Fructose (mM)	Sodium Pyruvate (mM)	Sodium DL-Lactate 60% (*w*/*w*) (mM)	NaCl (mM)	pH	Osmolarity (mOsm)
Experiment 1								
N		-	-	-	-	204.5	6.8	339
G		35	-	-	-	102.3	7.0	345
F		-	35	-	-	102.3	7.0	340
P		-	-	35	-	-	6.9	343
L		-	-	-	35	-	6.9	342
Experiment 2								
G70	H	70	-	-	-	-	6.8	323
G35/NaCl 18	H	35	-	-	-	17.5	7.0	342
G18/NaCl 9	H	17.5	-	-	-	8.8	6.9	321
G35	L	35	-	-	-	-	6.8	298
P35	L	-	-	35	-	-	6.9	303
P18	L	-	-	17.5	-	-	6.9	312
P18/NaCl 9	H	-	-	17.5	-	17.5	6.9	337
G35/P18	H	35	-	17.5	-	-	7.0	348
G18/P9	L	17.5	-	8.75	-	-	7.0	309

N: control (PBS + NaCl); G: glucose, F: fructose, P: pyruvate, L: lactate, G70, G35: extender containing glucose; G35/NaCl 18, G18/NaCl 9: extender containing glucose and NaCl; P35, P18: extender containing pyruvate; P18/NaCl9: extender containing pyruvate and NaCl; G35/P18, G18/P9: extender containing glucose and pyruvate. H: high-osmolarity group: 321–348 mOsm/kg vs.; L: low-osmolarity group: 298–313 mOsm/kg.

**Table 2:** The effects of NaCl supplementation, osmolarity, and energy substrate (extender) on sperm motility during storage at 17 °C.

		TM (%)	PM (%)	VCL (µm/s)	VAP (µm/s)	VSL (µm/s)	STR (%)	LIN (%)
NaCl supplementation								
	N	37.43 ± 1.64	95.21 ± 0.34	134.56 ± 1.86	92.54 ± 1.67	74.06 ± 1.83	81.42 ± 0.58	57.54 ± 0.91
	S	38.76 ± 2.23	95 ± 0.65	136.19 ± 2.6	92.14 ± 2.21	73.22 ± 2.57	80.65 ± 0.97	56.04 ± 1.39
Osmolarity								
	H	37.22 ± 1.79	95.02 ± 0.45	134.09 ± 2.07	91.65 ± 1.8	72.86 ± 2.01 *	81.15 ± 0.68	56.98 ± 1.01
	L	38.69 ± 1.97	95.3 ± 0.41	136.38 ± 2.21	93.35 ± 1.98	74.94 ± 2.21	81.19 ± 0.76	57.12 ± 1.17
Extender								
	G	36.7 ± 1.9	95.32 ± 0.4	134.92 ± 2.11	93.39 ± 1.77	75.33 ± 2	82.04 ± 0.64	58.56 ± 1.05
	M	36.49 ± 2.84	94.75 ± 0.66	133.27 ± 3.26	92.91 ± 2.71	74.11 ± 3.02	81.42 ± 1.08	58.58 ± 1.55
	P	40.36 ± 2.28	95.16 ± 0.63	136.58 ± 2.85	90.74 ± 2.69	71.5 ± 2.95	79.83 ± 1.01	53.99 ± 1.43

N: non-NaCl-supplemented; S: supplementation with NaCl; H: high osmolarity (321–348 mOsm/kg); L: low osmolarity (298–313 mOsm/kg); G: extender containing glucose; M: extender containing glucose and pyruvate; P: extender containing pyruvate. TM: total motility; PM: progressive motility; VCL: curvilinear velocity; VAP: average path velocity; VSL: straight-line velocity; STR: straightness; LIN: linearity of the curvilinear trajectory. (*) Within the same column indicates differences in pairwise comparisons (*p* < 0.05). The results are expressed as the mean ± standard error.

**Table 3:** The effects of NaCl supplementation, osmolarity, and energy substrate on quality parameters during sperm storage at 17 °C.

		Viability (%)	hMMP (%)	mROS (%)	Healthy (%)
NaCl supplementation					
	N	40.37 ± 1.16	41.55 ± 2.07	61.45 ± 1.44	34.29 ± 1.51
	S	38.73 ± 1.83	40.34 ± 2.85	63.11 ± 2.30	32.88 ± 2.46
Osmolarity					
	H	40.11 ± 1.31	42.07 ± 2.24	61.51 ± 1.62	34.79 ± 1.7
	L	39.45 ± 1.5	39.97 ± 2.53	62.64 ± 1.88	32.59 ± 2
Extender					
	G	33.18 ± 1.28 ^c^	35.73 ± 2.57 ^b^	69.93 ± 1.74 ^a^	24.98 ± 1.78 ^c^
	M	40.85 ± 1.64 ^b^	40.24 ± 3.36 ^b^	62.55 ± 2.09 ^b^	31.13 ± 2.07 ^b^
	P	47.87 ± 1.54 ^a^	48.87 ± 2.62 ^a^	51.23 ± 1.61 ^c^	47.23 ± 1.51 ^a^

N: non-NaCl-supplemented; S: supplementation with NaCl; H: high osmolarity (321–348 mOsm/kg); L: low osmolarity (298–313 mOsm/kg); G: extender containing glucose; M: extender containing glucose and pyruvate; P: extender containing pyruvate. hMMP: sperm with high mitochondrial membrane potential; mROS: mitochondrial reactive oxygen species production. Healthy: sperm having high mitochondrial membrane potential and low oxidative stress. ^a–c^ within the same column indicate differences in pairwise comparisons (*p* < 0.05). The results are expressed as the mean ± standard error.

**Table 4:** Significance levels of interaction factors for osmolarity, NaCl supplementation, and extender group on sperm quality and motility parameters during sperm storage at 17 °C.

	Viability	hMMP	mROS	Healthy	TM	PM	VCL	VAP	VSL	STR	LIN
Time × NaCl suppl	NS	NS	NS	NS	NS	NS	NS	NS	NS	NS	NS
Time × Osmolarity	NS	NS	NS	NS	NS	NS	NS	NS	*	NS	NS
Time × Extender	**	*	**	**	NS	NS	NS	NS	NS	NS	NS
Time × NaCl suppl × Osmolarity	NS	NS	NS	NS	NS	NS	NS	NS	NS	NS	NS
Time × NaCl suppl × Extender	NS	NS	NS	NS	NS	NS	NS	NS	NS	NS	NS
Time × Osmolarity × Extender	NS	NS	NS	NS	NS	NS	NS	NS	NS	NS	NS

NaCl Suppl: extender supplementation with NaCl; hMMP: sperm with high mitochondrial membrane potential (%); mROS: mitochondrial reactive oxygen species production (%); Healthy: sperm having high mitochondrial membrane potential and low oxidative stress (%); TM: total motility (%); PM: progressive motility (%); VAP: average path velocity (μm/s); VCL: curvilinear velocity (μm/s); VSL: straight-line velocity (μm/s); STR: straightness (%); LIN: linearity of the curvilinear trajectory (%). NS: no significant differences; * *p* < 0.05; ** *p* < 0.001.

**Table 5:** The effect of glucose or pyruvate concentration on in vitro sperm quality and motility parameters when stored for long periods at 17 °C in liquid form.

	Glucose			Pyruvate	
	18	35	70	18	35
Sperm quality parameters					
Viability (%)	40.07 ± 1.16 ^a^	33.98 ± 1.32 ^ab^	31.93 ± 2.05 ^b^	52.12 ± 1.95 ^a^	40.29 ± 4.37 ^b^
hMMP (%)	40.89 ± 1.19 ^b^	30.91 ± 1.86 ^c^	54.43 ± 6.24 ^a^	36.86 ± 3.82 ^b^	42.47 ± 5.76 ^a^
mROS (%)	59.83 ± 1.14 ^c^	62.98 ± 1.31 ^b^	74.75 ± 3.08 ^a^	48.13 ± 2.45 ^b^	60.58 ± 5.14 ^a^
Healthy (%)	99.36 ± 0.2 ^a^	90.38 ± 2.65 ^b^	64.93 ± 5.93 ^c^	87.47 ± 3.39 ^a^	73.84 ± 5.74 ^b^
Sperm motility parameters					
TM (%)	31.78 ± 3.03 ^b^	48.81 ± 2.47 ^a^	20.79 ± 2.71 ^c^	34.98 ± 2.31 ^b^	56.53 ± 3.81 ^a^
PM (%)	93.52 ± 1.37 ^b^	96.57 ± 0.38 ^a^	94.22 ± 0.65 ^b^	93.91 ± 0.96 ^b^	97.04 ± 0.42 ^a^
VCL (µm/s)	141.57 ± 4.43 ^b^	149.78 ± 3.13 ^a^	112.76 ± 2.56 ^c^	133.06 ± 3.28 ^b^	140.74 ± 3.25 ^a^
VAP (µm/s)	93.83 ± 4.75 ^b^	103.58 ± 2.75 ^a^	79.96 ± 2.8 ^c^	87.66 ± 2.92 ^b^	101.15 ± 1.79 ^a^
VSL (µm/s)	73.06 ± 5.95 ^b^	84.94 ± 2.75 ^a^	63.22 ± 2.15 ^c^	67.22 ± 3.75 ^b^	83 ± 1.77 ^a^
STR (%)	80.42 ± 2.06 ^b^	80.6 ± 0.73 ^b^	84.85 ± 0.59 ^a^	79.32 ± 1.43 ^a^	80.89 ± 0.75 ^a^
LIN (%)	53.78 ± 2.87 ^c^	57.54 ± 1.17 ^b^	61.61 ± 1.37 ^a^	53.2 ± 1.97 ^b^	60.64 ± 1.37 ^a^

hMMP: sperm with high mitochondrial membrane potential; mROS: mitochondrial reactive oxygen species production; Healthy: sperm having high mitochondrial membrane potential and low oxidative stress; TM: total motility; PM: progressive motility; VCL: curvilinear velocity; VAP: average path velocity; VSL: straight-line velocity; STR: straightness; LIN: linearity of the curvilinear trajectory. (^a–c^) indicate differences between concentrations inside the same energy substrate extender (*p* < 0.05).

## Data Availability

The data in this study can be obtained from the corresponding author upon request.

## Supplementary Materials

The following supporting information can be downloaded at https://www.mdpi.com/article/10.3390/biology14070782/s1. Table S1: The effects of experimental groups G35, G70, P35, P18, G35/P18, G18/P9, G35/N18, G18/N9, and P18/N9 on motility during refrigerated storage at 17 °C; Table S2: The eeffects of experimental groups G35, G70, P35, P18, G35/P18, G18/P9, G35/N18, G18/N9, and P18/N9 on spermatozoa viability, mitochondrial membrane potential, and oxidative stress reaction, and healthy population when stored at 17 °C.

## References

[1] Leboeuf B., Guillouet P., Batellier F., Bernelas D., Bonné J.L., Forgerit Y., Renaud G., Magistrini M. (2003). Effect of native phosphocaseinate on the in vitro preservation of fresh semen. Theriogenology.

[2] Mocé E., Mocé M.L., Lozano-Palazón S.A., Bernácer J., Martínez-Granell M.M., Esteve I.C., Bernat F., Contreras S.J., Villalba I., Gómez E.A. (2022). Fertility prediction in dairy goats from Murciano-Granadina breed: The role of sperm evaluation and female traits. Animal.

[3] Leboeuf B., Restall B., Salamon S. (2000). Production and storage of goat semen for artificial insemination. Anim. Reprod. Sci..

[4] Peña F.J., Ortiz-Rodríguez J.M., Gaitskell-Phillips G.L., Gil M.C., Ortega-Ferrusola C., Martín-Cano F.E. (2022). An integrated overview on the regulation of sperm metabolism (glycolysis-Krebs cycle-oxidative phosphorylation). Anim. Reprod. Sci..

[5] Peña F.J., Gibb Z. (2022). Oxidative stress and reproductive function: Oxidative stress and the long-term storage of horse spermatozoa. Reproduction.

[6] Du Plessis S.S., Agarwal A., Mohanty G., Van Der Linde M. (2015). Oxidative phosphorylation versus glycolysis: What fuel do spermatozoa use?. Asian J. Androl..

[7] Hernández-Avilés C., Ramírez-Agámez L., Love C.C., Friedrich M., Pearson M., Kelley D.E., Beckham A.M.N., Teague S.R., LaCaze K.A., Brinsko S.P. (2021). The effects of metabolic substrates glucose, pyruvate, and lactate added to a skim milk-based semen extender for cooled storage of stallion sperm. Theriogenology.

[8] Setiawan R., Christi R.F., Alhuur K.R.G., Widyastuti R., Solihati N., Rasad S.D., Hidajat K., Do D.N. (2024). Impact of glucose and pyruvate on adenosine triphosphate production and sperm motility in goats. Anim. Biosci..

[9] Miki K. (2007). Energy metabolism and sperm function. Soc. Reprod. Fertil. Suppl..

[10] Qiu J., Li Y., Xie H., Li Q., Dong H., Sun M. (2016). Effects of glucose metabolism pathways on sperm motility and oxidative status during long-term liquid storage of goat semen. Theriogenology.

[11] Chunrong L., Larbi A., Wu G., Hong Q., Quan G. (2019). Improving the quality of cryopreserved goat semen with a commercial bull extender supplemented with resveratrol. Anim. Reprod. Sci..

[12] Mocé E., Lozano-Palazón S.A., Martínez-Granell M.D.M., Mocé M.L., Gómez E.A. (2020). Effect of the Refrigeration System on In Vitro Quality and In Vivo Fertility of Goat Buck Sperm. Animals.

[13] Gororo E., Zulu P.T., Chatiza F.P., Mhuka C. (2019). Effects of different extenders and storage temperatures on longevity of small East African goat (*Capra hircus*) semen. Small Rumin. Res..

[14] Roca J., Carrizosa J.A., Campos I., Lafuente A., Vazquez J.M., Martinez E. (1997). Viability and fertility of unwashed Murciano-Granadina goat spermatozoa diluted in Tris-egg yolk extender and stored at 5 °C. Small Rumin. Res..

[15] Xu C., Zhou J., Zhao B., Lan G., Luo M., Chang Z., Sui H., Tan J. (2009). Liquid Storage of Goat Semen in Chemically Defined Extenders. Reprod. Domest. Anim..

[16] Silvestre M.A., Sánchez J.P., Gómez E.A. (2004). Vitrification of goat, sheep, and cattle skin samples from whole ear extirpated after death and maintained at different storage times and temperatures. Cryobiology.

[17] Konyali C., Tomás C., Blanch E., Gómez E.A., Graham J.K., Mocé E. (2013). Optimizing conditions for treating goat semen with cholesterol-loaded cyclodextrins prior to freezing to improve cryosurvival. Cryobiology.

[18] Gacem S., Castello-Ruiz M., Hidalgo C.O., Tamargo C., Santolaria P., Soler C., Yániz J.L., Silvestre M.A. (2023). Bull Sperm SWATH-MS-Based Proteomics Reveals Link between High Fertility and Energy Production, Motility Structures, and Sperm-Oocyte Interaction. J. Proteome Res..

[19] Varner D.D., Blanchard T.L., Love C.L., Garcia M.C., Kenney R.M. (1987). Effects of semen fractionation and dilution ratio on equine spermatozoal motility parameters. Theriogenology.

[20] Batista M., Niño T., Alamo D., Castro N., Santana M., González F., Cabrera F., Gracia A. (2009). Successful artificial insemination using semen frozen and stored by an ultrafreezer in the Majorera goat breed. Theriogenology.

[21] Gangwar C., Kharche S.D., Ranjan R., Kumar S., Goel A.K., Jindal S.K., Agrawal S.K. (2015). Effect of vitamin C supplementation on freezability of Barbari buck semen. Small Rumin. Res..

[22] Naing S.W., Wahid H., Mohd Azam K., Rosnina Y., Zuki A.B., Kazhal S., Bukar M.M., Thein M., Kyaw T., San M.M. (2010). Effect of sugars on characteristics of Boer goat semen after cryopreservation. Anim. Reprod. Sci..

[23] Ritar A.J., Salam S. (1983). Fertility of fresh and frozen -thawed semen of the angora goat. Aust. J. Biol. Sci..

[24] Salvador I., Viudes-De-Castro M.P., Bernacer J., Gómez E.A., Silvestre M.A. (2005). Factors affecting pregnancy rate in artificial insemination with frozen semen during non-breeding season in Murciano-Granadina goats: A field assay. Reprod. Domest. Anim..

[25] Salvador I., Yániz J., Viudes-de-Castro M.P., Gómez E.A., Silvestre M.A. (2006). Effect of solid storage on caprine semen conservation at 5 °C. Theriogenology.

[26] Nunes J.F., Corteel J.-M., Combarnous Y., Baril G., Leboeuf B. (1982). Rôle du plasma séminal dans la survie in vitro des spermatozoïdes de bouc. Reprod. Nutr. Dév..

[27] Galián S., Peinado B., Almela L., Poto Á., Ruiz S. (2023). Post-Thaw Quality of Spermatozoa Frozen with Three Different Extenders in the Murciano Granadina Goat Breed. Animals.

[28] Sadeghi S., Del Gallego R., García-Colomer B., Gómez E.A., Yániz J.L., Gosálvez J., López-Fernández C., Silvestre M.A. (2020). Effect of sperm concentration and storage temperature on goat spermatozoa during liquid storage. Biology.

[29] Amaral A. (2022). Energy metabolism in mammalian sperm motility. WIREs Mech. Dis..

[30] Van de Hoek M., Rickard J.P., de Graaf S.P. (2024). Manipulation of metabolism to improve liquid preservation of mammalian spermatozoa. Anim. Reprod. Sci..

[31] Tsujii H., Ohta E., Miah A.G., Hossain S., Salma U. (2006). Effect of fructose on motility, acrosome reaction and in vitro fertilization capability of boar spermatozoa. Reprod. Med. Biol..

[32] Sun W., Jiang S., Su J., Zhang J., Bao X., Ding R., Shi P., Li S., Wu C., Zhao G. (2021). The effects of cryopreservation on the acrosome structure, enzyme activity, motility, and fertility of bovine, ovine, and goat sperm. Anim. Reprod..

[33] Tourmente M., Villar-Moya P., Rial E., Roldan E.R.S. (2015). Differences in ATP generation via glycolysis and oxidative phosphorylation and relationships with sperm motility in mouse species. J. Biol. Chem..

[34] Reynolds S., Ismail N.F.B., Calvert S.J., Pacey A.A., Paley M.N.J. (2017). Evidence for Rapid Oxidative Phosphorylation and Lactate Fermentation in Motile Human Sperm by Hyperpolarized 13 C Magnetic Resonance Spectroscopy. Sci. Rep..

[35] Hereng T.H., Elgstøen K.B.P., Cederkvist F.H., Eide L., Jahnsen T., Sklhegg B.S., Rosendal K.R. (2011). Exogenous pyruvate accelerates glycolysis and promotes capacitation in human spermatozoa. Hum. Reprod..

[36] Ramírez-Agámez L., Hernández-Avilés C., Ortíz I., Love C.C., Varner D.D., Hinrichs K. (2023). Lactate as the sole energy substrate induces spontaneous acrosome reaction in viable stallion spermatozoa. Andrology.

[37] Becerro-Rey L., Martín-Cano F.E., Ferrusola C.O., Rodríguez-Martínez H., Gaitskell-Phillips G., da Silva-Álvarez E., Silva-Rodríguez A., Gil M.C., Peña F.J. (2024). Aging of stallion spermatozoa stored in vitro is delayed at 22 °C using a 67 mm glucose-10 mm pyruvate-based media. Andrology.

[38] Atashfaraz E., Farokhi F., Najafi G. (2013). Protective Effect of Ethyl Pyruvate on Epididymal Sperm Characteristics, Oxidative Stress and Testosterone Level in Methotrexate Treated Mice. J. Reprod. Infertil..

[39] Thompson J.A., Love C.C., Stich K.L., Brinsko S.P., Blanchard T.L., Varner D.D. (2004). A Bayesian approach to prediction of stallion daily sperm output. Theriogenology.

[40] Varma S.D., Devamanoharan P.S., Morris S.M. (1990). Photoinduction of cataracts in rat lens in vitro. Preventive effect of pyruvate. Exp. Eye Res..

[41] De Lamirande E., Gagnon C. (1993). Impact of reactive oxygen species on spermatozoa: A balancing act between beneficial and detrimental effects. Hum. Reprod..

[42] Andrae U., Singh J., Ziegler-Skylakakis K. (1985). Pyruvate and related α-ketoacids protect mammalian cells in culture against hydrogen peroxide-induced cytotoxicity. Toxicol. Lett..

[43] Brand K.A., Hermfisse U. (1997). Aerobic glycolysis by proliferating cells: A protective strategy against reactive oxygen species. FASEB J..

[44] Foutouhi A., Meyers S. (2022). Comparative oxidative metabolism in mammalian sperm. Anim. Reprod. Sci..

[45] Darr C.R., Varner D.D., Teague S., Cortopassi G.A., Datta S., Meyers S.A. (2016). Lactate and pyruvate are major sources of energy for stallion sperm with dose effects on mitochondrial function, motility, and ROS production. Biol. Reprod..

[46] Upreti G.C., Jensen K., Munday R., Duganzich D.M., Vishwanath R., Smith J.F. (1998). Studies on aromatic amino acid oxidase activity in ram spermatozoa: Role of pyruvate as an antioxidant. Anim. Reprod. Sci..

[47] Sattar A., Farooq M., Khan M., Rehman A., Javed K. (2018). 43 Effect of addition of sodium pyruvate in extender on post-thaw quality of Beetal buck semen. J. Anim. Sci..

[48] Korkmaz F., Malama E., Siuda M., Leiding C., Bollwein H. (2017). Effects of sodium pyruvate on viability, synthesis of reactive oxygen species, lipid peroxidation and DNA integrity of cryopreserved bovine sperm. Anim. Reprod. Sci..

[49] Gibb Z., Lambourne S.R., Quadrelli J., Smith N.D., Aitken R.J. (2015). L-carnitine and pyruvate are prosurvival factors during the storage of stallion spermatozoa at room temperature. Biol. Reprod..

[50] Breininger E., Beconi M.T. (2014). Ascorbic acid or pyruvate counteracts peroxidative damage in boar sperm cryopreserved with or without alpha-tocopherol. Anim. Sci. Pap. Rep..

[51] Bruemmert J.E., Coy R.C., Squires E.L., Graham J.K. (2002). Effect of pyruvate on the function of stallion spermatozoa stored for up to 48 hours. J. Anim. Sci..

[52] Groussard C., Morel I., Chevanne M., Monnier M., Cillard J., Delamarche A. (2000). Free radical scavenging and antioxidant effects of lactate ion: An in vitro study. J. Appl. Physiol..

[53] Hugentobler S.A., Humpherson P.G., Leese H.J., Sreenan J.M., Morris D.G. (2008). Energy substrates in bovine oviduct and uterine fluid and blood plasma during the oestrous cycle. Mol. Reprod. Dev..

[54] Ortiz-Rodríguez J.M., Martín-Cano F.E., Gaitskell-Phillips G.L., Silva A., Ortega-Ferrusola C., Gil M.C., Peña F.J. (2021). Low glucose and high pyruvate reduce the production of 2-oxoaldehydes, improving mitochondrial efficiency, redox regulation, and stallion sperm function. Biol. Reprod..

[55] Li Y., Zhang G., Wen F., Xian M., Guo S., Zhang X., Feng X., Hu Z., Hu J. (2023). Glucose Starvation Inhibits Ferroptosis by Activating the LKB1/AMPK Signaling Pathway and Promotes the High Speed Linear Motility of Dairy Goat Sperm. Animals.

[56] Martín-Cano F.E., Gaitskell-Phillips G., Becerro-Rey L., da Silva E., Masot J., Redondo E., Silva-Rodríguez A., Ortega- Ferrusola C., Gil M.C., Peña F.J. (2024). Pyruvate enhances stallion sperm function in high glucose media improving overall metabolic efficiency. Theriogenology.

[57] Johnson L.A., Weitze K.F., Fiser P., Maxwell W.M.C. (2000). Storage of boar semen. Anim. Reprod. Sci..

[58] Storey B.T. (2008). Mammalian sperm metabolism: Oxygen and sugar, friend and foe. Int. J. Dev. Biol..

[59] Mortimer S.T. (1997). A critical review of the physiological importance and analysis of sperm movement in mammals. Hum. Reprod. Update.

[60] Dorado J., Molina I., Muñoz-Serrano A., Hidalgo M. (2010). Identification of sperm subpopulations with defined motility characteristics in ejaculates from Florida goats. Theriogenology.

[61] Purdy P.H. (2006). A review on goat sperm cryopreservation. Small Rumin. Res..

