# Supplementary material for: The Effects of Extender Energetic Substrate Type on Goat Sperm Stored at 17 °C
Source: Biology (Basel). 2025 Jun 27;14(7):782. doi: 10.3390/biology14070782 (PMC12292228; doi:10.3390/biology14070782)
Supplement: Supplementary file 1 [file biology-14-00782-s001.zip › biology-3693416-supplementary.pdf]

Table S1. The effects of experimental groups G35, G70, P35, P18, G35/P18, G18/P9, G35/N18, G18/N9 and P18/N9 on motility during refrigerated storage at 17 °C.

	Extender	TM (%)	PM (%)	VCL (μm/s)	VAP (μm/s)	VSL (μm/s)	STR (%)	LIN (%)
**Oh**	G18/N9	46.48 ± 15.31	96.84 ± 1.63	151.84 ± 13.48[abc]	104.77 ± 13.62[ab]	87.67 ± 14.96[ab]	82.17 ± 5.59	58.05 ± 6.14
	G18/P18	47.58 ± 19.64	95.8 ± 4.72	153.54 ± 16.37[ab]	111.06 ± 14.4[a]	92.67 ± 17.96[a]	81.57 ± 8.5	60.96 ± 9.92
	G35	44.73 ± 17.8	97.18 ± 1.48	153.86 ± 16.71[ab]	107.91 ± 16.56 [ab]	90.83 ± 17.19[ab]	83.23 ± 3.31	59.72 ± 6.35
	G35/18	41.99 ± 14.14	97.04 ± 1.57	151.55 ± 18.77[abcd]	105.44 ± 10.99 [ab]	88.33 ± 11.18[ab]	82.23 ± 4.51	59.08 ± 3.63
	G35/P18	46.05 ± 12.66	96.73 ± 1.84	142.4 ± 24.56[abcde]	100.56 ± 20.34[abc]	84.83 ± 20.52[abc]	83.02 ± 3.79	60.37 ± 7.13
	G70	51.82 ± 16.41	97.88 ± 0.85	152.38 ± 13.68[abc]	107.49 ± 12.38 [ab]	91.33 ± 11.27[a]	83.14 ± 1.89	60.02 ± 4.48
	P18	47.7 ± 14.17	97.58 ± 0.97	156.93 ± 10.62[a]	108.06 ± 16.7 [ab]	90.33 ± 16.03[ab]	82.59 ± 4.36	58 ± 7.1
	P18/9	49.34 ± 15.01	97.25 ± 1.85	153.75 ± 14.17[ab]	106.93 ± 14.41[ab]	88 ± 16.59[ab]	80.77 ± 5.54	57.81 ± 8.2
	P35	47.33 ± 18.98	97.61 ± 0.93	155.64 ± 13.75[a]	108.53 ± 17[ab]	91.17 ± 17.27[a]	83.02 ± 4.47	59.19 ± 6.95
**24h**	G18/N9	43.43 ± 16.9	95.97 ± 2.26	132.85 ± 15.15 [abcde]	93.67 ± 13.24[abcd]	76.33 ± 14.08[abcd]	82.24 ± 4.84	60.02 ± 8.16
	G18/P18	35.08 ± 17.27	95.17 ± 2.51	128.64 ± 9.88 [abcde]	91.35 ± 11.98 [abcd]	73.17 ± 14.08 [abcd]	81.73 ± 6.12	60.09 ± 10.34
	G35	37.95 ± 12.27	96.63 ± 1.26	124.1 ± 9.49 [abcde]	87.83 ± 14.01 [abcd]	73 ± 14.68 [abcd]	83.97 ± 4.38	61.1 ± 8.72
	G35/18	38.17 ± 18.16	96.17 ± 2.29	127.63 ± 5.79 [abcde]	88.89 ± 11.69 [abcd]	73 ± 13.25 [abcd]	83.52 ± 5.28	59.66 ± 9.29

	G35/P18	34.3 ± 14.25	94.99 ± 2.47	125.49 ± 5.8 ^{abcde}	86.42 ± 10.73 ^{abcd}	68.83 ± 12.06 ^{abcd}	81.41 ± 5.27	58.18 ± 9.17
	G70	34.36 ± 13.26	95.39 ± 3.21	129.75 ± 5.76 ^{abcde}	93.58 ± 13.52 ^{abcd}	76 ± 17.16 ^{abcd}	82.05 ± 6.81	61.1 ± 11.29
	P18	41 ± 18.13	95.67 ± 1.3	139.28 ± 9.38 ^{abcde}	94.84 ± 11.04 ^{abcd}	76.17 ± 11.32 ^{abcd}	80.4 ± 4.91	56.29 ± 8.39
	P18/N9	42.98 ± 12.49	96.05 ± 1.51	138.88 ± 16.36 ^{abcde}	92.87 ± 13.35 ^{abcd}	76 ± 13.46 ^{abcd}	81 ± 7.05	56.67 ± 11.37
	P35	43.94 ± 19.77	95.75 ± 2.27	133.5 ± 14.21 ^{abcde}	91.16 ± 14.42 ^{abcd}	73 ± 15.31 ^{abcd}	80.08 ± 5.95	56.73 ± 9.3
**48h**	G18/N9	33.83 ± 18.61	92.8 ± 5.97	131.36 ± 12.97^{abcd}	84.41 ± 6.75 ^{abcd}	61.67 ± 15.31 ^{abcd}	77.51 ± 9.12	51.7 ± 15.14
	G18/P18	34.23 ± 18.25	94.38 ± 4.7	125.89 ± 18.13 ^{abcde}	84.93 ± 10.73 ^{abcd}	66.67 ± 13.63 ^{abcd}	81.22 ± 8.94	56.61 ± 11.15
	G35	22.7 ± 8.81	92.99 ± 3.82	122.06 ± 14.4 ^{bcde}	81.49 ± 9.17^{bcd}	62 ± 10.26 ^{abcd}	80.93 ± 7.18	56.39 ± 10.99
	G35/N18	25.42 ± 14.39	92.66 ± 3.98	119.74 ± 13.24^{cde}	79.89 ± 8.86^{bcd}	60.67 ± 9.5 ^{abcd}	82.06 ± 4.01	56.48 ± 7.48
	G35/P18	21.72 ± 11.06	91.42 ± 5.34	123.67 ± 21.79 ^{abcde}	83.15 ± 12.02 ^{abc}d	58.5 ± 9.09^{bcd}	79.56 ± 7.32	55.25 ± 10.57
	G70	19.55 ± 8.51	92.35 ± 3.89	121.89 ± 13.96 ^{bcde}	85.36 ± 10.66 ^{abcd}	63.17 ± 12.12 ^{abcd}	81.38 ± 6.92	59.46 ± 12.29
	P18	29.61 ± 13.56	92.58 ± 4.33	116.19 ± 15.31^{e}	69.84 ± 8.85^{d}	48.83 ± 12.5^{d}	76.68 ± 8.62	46.48 ± 11.93
	P18/N9	27.22 ± 13.58	90.2 ± 10.59	118.17 ± 21.71^{de}	72.37 ± 16.3^{cd}	47.33 ± 17.58^{d}	74.32 ± 13.38	44.86 ± 13.82
	P35	34.15 ± 17.46	93.78 ± 4.72	116.86 ± 13.89^{e}	72.09 ± 10.07^{cd}	52.67 ± 11.91^{cd}	79.65 ± 8.81	49.86 ± 10.31

Goat sperm were stored in PBS containing glucose (G) or pyruvate (P) and supplemented with NaCl (N). Motility parameters were recorded by CASA at 2h, 24h and 48h of incubation at 17 °C. Total motility (TM), Progressive motility (PM). Curvilinear velocity (VCL). Average Path Velocity (VAP), Straight Line velocity (VSL), Straightness index (STR) and lineariry (LIN). $^{(a–e)}$ indicate differences between extender group during 48h (p< 0.05). Results are expressed as mean ± standard error.

Table S2. The effects of experimental groups G35, G70, P35, P18, G35/P18, G18/P9, G35/N18, G18/N9 and P18/N9 on spermatozoa viability, mitochondria membrane potential and oxidative stress reaction and healthy population when stored at 17 °C.

Time	Extender group	Viability (%)	hMMP (%)	mROS (%)	Healthy (%)
**0h**	G18/N9	42.68 ± 5.39 [abcd]	42.93 ± 6.41	55.98 ± 5.83 [abc]	99.75 ± 0.06[a]
	G18/P18	41.18 ± 2.76 [abcd]	41.52 ± 4.82	58.33 ± 2.79 [abc]	99.33 ± 0.16[a]
	G35	39.95 ± 4.4 [abcd]	37.27 ± 3.69	59.12 ± 4.66 [abc]	99.5 ± 0.12[a]
	G35/N18	40 ± 4.47 [abcd]	37.15 ± 3.37	58.65 ± 4.83 [abc]	99.63 ± 0.05[a]
	G35/P18	40.53 ± 4.27 [abcd]	46.32 ± 7.27	58.55 ± 4.63 [abc]	98.77 ± 0.5[a]
	G70	39.7 ± 3.62 [abcd]	35.43 ± 2.74	59.32 ± 3.92[abc]	99.73 ± 0.07[a]
	P18	46.87 ± 4.14[abc]	41.52 ± 4.34	51.85 ± 4.61 [abc]	98.78 ± 0.67[a]
	P18/N9	47.35 ± 2.88[ab]	48.87 ± 4.84	51.48 ± 2.96[bc]	98.65 ± 0.74[a]
	P35	44.98 ± 3.71[abcd]	46.5 ± 5.52	54.58 ± 3.94 [abc]	98.3 ± 0.71[a]
**24h**	G18/N9	35.25 ± 2.31 [abcd]	33.1 ± 9.31	76.55 ± 4.35abc	51.67 ± 8.25[cd]
	G18/P18	44.68 ± 5.07 [abcd]	39.37 ± 10.81	62.7 ± 6.13abc	69.13 ± 8.56[abcd]
	G35	32.94 ± 2.31 [abcd]	45.22 ± 14.04	76.94 ± 5.16abc	57.88 ± 11.73[cd]
	G35/N18	30.47 ± 5.49 [abcd]	33.58 ± 11.26	80.37 ± 5.07a	42.42 ± 8.79[d]
	G35/P18	49.02 ± 4.93[a]	41.1 ± 10.38	56.52 ± 5.97 abc	76.55 ± 8.5[abc]

	G70	36.05 ± 1.64 ^{abcd}	37.93 ± 10.73	74.43 ± 4.67abc	59.08 ± 10.18^{cd}
	P18	52.13 ± 6.88^{a}	38.58 ± 11.23	47.28 ± 7.13^{c}	97.3 ± 1.02^{ab}
	P18/N9	50.27 ± 5.75^{a}	41.82 ± 9.37	48.88 ± 5.92b^{c}	98.23 ± 0.85^{a}
	P35	46.58 ± 5.33^{abc}	49.72 ± 12.34	53.58 ± 5.69 ^{abc}	97.6 ± 1.29^{a}
**48h**	G18/9	30.3 ± 6.09 ^{abcd}	37.97 ± 11.1	70.27 ± 7.43^{abc}	65.85 ± 8.6^{bcd}
	G18/P18	32.65 ± 0.87 ^{abcd}	34.23 ± 8.26	69.42 ± 3.85^{abc}	59.08 ± 7.68^{cd}
	G35	24.55 ± 1.96^{bcd}	28.05 ± 10.69	75.67 ± 5.77^{abc}	54.13 ± 6.9^{cd}
	G35/N18	24.18 ± 1.28^{cd}	30.35 ± 11.37	75.85 ± 5.37^{abc}	58.07 ± 5.3^{cd}
	G35/P18	37.02 ± 1.83 ^{abcd}	38.9 ± 9.41	69.78 ± 5.64^{abc}	61.78 ± 11.58^{cd}
	G70	22.08 ± 0.85^{d}	31.3 ± 11.89	77.13 ± 5.16^{ab}	62.05 ± 5.78^{cd}
	P18	49.18 ± 5.2^{a}	60.82 ± 7.17	49.9 ± 5.38^{bc}	98.13 ± 0.54^{a}
	P18/N9	48.07 ± 4.62^{a}	57.27 ± 5.52	50 ± 4.48^{bc}	98.87 ± 0.36^{a}
	P35	45.4 ± 4.67^{abc}	54.77 ± 6.59	53.52 ± 4.91^{abc}	98.63 ± 0.43^{a}

Goat sperm were stored in PBS containing glucose (G) or pyruvate (P) and supplemented or not with NaCl (N). Sperm with high mitochondrial membrane potential (hMMP), Reactive oxygen species production (mROS), Sperm having high mitochondrial membrane potential and low oxidative stress (Healthy). (a–d) indicate differences between extender group during 48h (p< 0.05). Results are expressed as mean ± standard error.
